# Neutrophils, not macrophages, aid phage-mediated control of pulmonary *Pseudomonas aeruginosa* infection

**DOI:** 10.3389/fimmu.2025.1681461

**Published:** 2025-11-19

**Authors:** Chantal Weissfuss, Karen Hoffmann, Ulrike Behrendt, Magdalena Bürkle, Shailey G. Twamley, Imke H. E. Korf, Katharina Ahrens, Christine Rohde, Christian M. Zobel, Laurent Debarbieux, Jean-Damien Ricard, Martin Witzenrath, Geraldine Nouailles

**Affiliations:** 1 Charité - Universitätsmedizin Berlin, Corporate Member of Freie Universität Berlin and Humboldt-Universität zu Berlin, Department of Infectious Diseases, Respiratory Medicine and Critical Care, Berlin, Germany; 2 Department of Internal Medicine, Bundeswehrkrankenhaus Berlin, Berlin, Germany; 3 Charité - Universitätsmedizin Berlin, Corporate Member of Freie Universität Berlin and Humboldt-Universität zu Berlin, Institute of Functional Anatomy, Berlin, Germany; 4 Pharmaceutical Biotechnology, Fraunhofer Institute for Toxicology and Experimental Medicine, Braunschweig, Germany; 5 Leibniz Institute DSMZ - German Collection of Microorganisms and Cell Cultures, Braunschweig, Germany; 6 Institut Pasteur, Université Paris Cité, CNRS UMR6047, Department of Microbiology, Bacteriophage Bacteria Host Laboratory, Paris, France; 7 Université Paris Cité, Inserm, UMR 1137, Infection Antimicrobials Modelling Evolution, Paris, France; 8 Assistance Publique-Hôpitaux de Paris, Hôpital Louis Mourier, DMU ESPRIT, Médecine Intensive Réanimation, Colombes, France; 9 German Center for Lung Research (DZL), Berlin, Germany

**Keywords:** phage therapy, immunophage synergy, pneumonia, innate immunity, *Pseudomonas aeruginosa*

## Abstract

**Background:**

The increasing prevalence of multidrug-resistant (MDR) bacteria has reduced the effectiveness of standard antibiotics, prompting renewed interest in bacteriophage (phage) therapy as an alternative or adjunctive treatment. Phage therapy offers high specificity, self-amplification at infection sites, and minimal disruption to the gut microbiota. However, clinical implementation is challenging, due to the risk of phage resistance and uncertainties regarding optimal dosing and immune interactions.

**Methods:**

Previously, we demonstrated that a two-phage cocktail exhibited low immunogenicity in mice and, when combined with meropenem, significantly improved clearance of ventilator-associated *Pseudomonas aeruginosa* pneumonia, reduced inflammation, and disrupted biofilms more effectively than either treatment alone. In the present study, we investigated the interplay between this phage cocktail and innate immune defenses using a murine respiratory infection model and human *in vitro* assays.

**Results:**

Our findings reveal that the therapeutic efficacy of phage treatment is critically dependent on the presence of neutrophils, which act synergistically with phages to achieve effective bacterial clearance, particularly when bacterial burden exceeds a defined threshold. Alveolar macrophages, however, do not significantly contribute to infection resolution *in vivo*.

**Conclusion:**

Since neutrophils play a key-role in supporting phage-mediated *Pseudomonas* clearance, the efficacy of phage therapy is closely linked to the hosts immune competence – an important consideration when treating immunocompromised patients.

## 1 Introduction

The global rise of multidrug-resistant (MDR) bacterial pathogens represents a public health threat and dramatically limits the efficacy of conventional antibiotic therapies (1, 2). In response, bacteriophage (phage) therapy has re-emerged as a promising alternative or adjunctive therapeutic approach. Compared to antibiotics, phage therapy offers several key advantages: phages are highly specific to their bacterial hosts, capable of self-amplification at the site of infection, and, unlike broad-spectrum antibiotics, do not adversely affect the gut microbiota (3, 4). Despite these benefits, there are still different concerns regarding the clinical application of phages (5). Therapeutic efficacy is influenced by the type and concentration of phages used; as monotherapy can promote the emergence of phage-resistant bacterial clones, personalized phage cocktails or combinations with antibiotics for example, are recommended to enhance treatment outcomes (6).

Beyond their bactericidal activity, phages are increasingly recognized as immunomodulatory agents capable of influencing host inflammatory responses and of modulating the outcome of infection. Previous investigations have shown that phages can interact directly with phagocytes, including neutrophils and macrophages, without impairing their antimicrobial functions, supporting their respective safety as therapeutic agents (7). Cafora et al. demonstrated a phage cocktail targeting *Pseudomonas aeruginosa* showed anti-inflammatory effects in zebrafish embryos with CFTR-loss-of-function (as a model of cystic fibrosis, CF), reducing pro-inflammatory markers even without bacterial infection. They attributed the immune-modulatory effect to phage virion proteins that activate the Toll-like Receptor (TLR) pathway. In CFTR-deficient zebrafish, this activation by phage injection also resulted in reduced neutrophil migration after acute inflammation (8). Similarly, phages in combination with phosphatidylserine/phosphatidic acid liposomes were shown to enhance macrophage phagocytic activity and to reduce bacterial burden in infected CFTR-deficient models, further underscoring the immunomodulatory potential of phage therapy (9).

Although studies show that phages only minimally impact the host microbiome and immune responses, their interaction with the host immune system during bacterial infections remains poorly understood. The distinct roles of innate immune cells, particularly neutrophils and alveolar macrophages in shaping the outcomes of phage therapy in the lung are not well defined, representing a significant gap in current knowledge. In that context, *immunophage synergy* describes the cooperative action between bacteriophages and the innate immune system, emphasizing that immune effector cell functions are integral to therapeutic success. Experimental evidence supports this concept: host immune responses can directly influence the effectiveness of phages to clear bacterial infections *in vivo*. For example, Roach et al. demonstrated that successful phage therapy against acute pneumonia caused by MDR *P. aeruginosa* in mice relied on synergistic interactions between therapeutic phages and neutrophil-mediated immunity, rather than on direct bacterial lysis alone (10).

Extracellular pathogens, such as *P. aeruginosa* are primarily targeted by type 3 immune responses, with neutrophils serving as the principal effector cells and (alveolar) macrophages recognizing lipopolysaccharide (LPS) from this Gram-negative bacterium to initiate innate immunity (11, 12). Neutrophils, along with other phagocytic cells, such as tissue-resident and recruited monocyte-derived macrophages, play a central role in the elimination of *Pseudomonas* through phagocytosis. Upon internalization, phagosomes mature into phagolysosomes, exposing the bacteria to potent antimicrobial mechanisms such as reactive oxygen and nitrogen species, and hydrolytic enzymes (13). However, *Pseudomonas* can resist these mechanisms for example via antioxidant enzymes or biofilm formation (1, 14). Hence, neutrophils, in particular, have emerged as key contributors to this mechanism, they are essential for the elimination of phage-resistant bacteria (10) and have been identified as key contributors to the success of phage therapy in murine models of intraperitoneal *P. aeruginosa* infection (15). Interestingly, while neutrophil involvement in phage efficacy is well established, recent data also suggest that macrophages may affect therapeutic outcomes by modulating phage persistence and distribution. For instance, macrophage-mediated clearance can reduce intrapulmonary phage density, thereby influencing bacterial killing dynamics (16).

Theoretical modeling has further elucidated the dynamics of phage–host immune interactions, suggesting that neither phages nor the innate immune system alone can eradicate bacterial infections (17). Instead, therapeutic success often relies on synergistic interactions: phages either reduce bacterial density to levels manageable by innate immune cells (static synergy), or disrupt bacterial populations in cycles that aid immune clearance of the pathogens (dynamic synergy) (17). However, phages with insufficient potency may fail, while overly aggressive immune responses can be counterproductive. Notably, modeling studies have identified a bacterial load threshold (6.94 log_10_ CFUeq/g) beyond which bacterial growth outpaces the capacity of the immune response without phage support (18). Phages can also contribute to infection control by disrupting bacterial defenses such as biofilms, thus re-exposing bacteria to immune clearance (17, 19). Clinical and experimental studies increasingly indicate that optimal phage therapy outcomes depend on a finely tuned balance between phage kinetics, bacterial burden, and innate immune activity (8, 20). Building on computational and mathematical models, recent work further demonstrates that variations in host immune strength and phage adsorption rates critically determine the threshold between therapeutic success and failure, with robust phage-immune synergy required to clear infections—especially in immunocompromised hosts, where moderately less effective phages or diminished immune function can dramatically decrease treatment efficacy (21). In terms of phage characteristics, those with low decay rates, high adsorption efficiency, and large burst sizes appear to be most effective (17). Nevertheless, even phages with lower adsorption rates may be therapeutically beneficial if they exhibit prolonged biological half-lives *in vivo*. Interestingly, a higher lysis rate can offset limitations in burst size (18).

In addition to modulating bacterial populations, phages may directly influence the immune system. While phages alone do not seem to activate neutrophils or trigger antimicrobial pathways in the absence of infection (22), the lysis of bacteria releases cell wall components and intracellular contents that may stimulate immune responses via pattern recognition receptor-dependent signaling pathways. Phages are also known to reside in the mucus layers of many animals, potentially serving as a first line of defense against mucosal bacterial invasion (23). Studies suggest that phages can cross epithelial barriers in the gut, come into direct contact with immune cells, and influence immune responses, even in the absence of bacterial co-stimulation (24–27). Consistent with these findings, phages of the Caudovirales order have been shown to prompt variable humoral responses in mice, including transient IgM and IgG production without strong systemic inflammation, indicating a generally tolerogenic immune profile, preventing excessive immune activation and tissue damage (28, 29). These results suggest phages trigger adaptive immunity without strong inflammatory effects, relevant for their therapeutic use.

We have previously demonstrated that a preclinical two-phage cocktail displayed limited immunogenicity in naive mice (29). The same cocktail significantly enhanced the therapeutic effectiveness of standard antibiotic treatment of *P. aeruginosa*-induced ventilator-associated pneumonia. When combined with phages, lower meropenem doses were sufficient to kill bacteria, while being associated with reduced inflammatory damage *in vivo* (30). *In vitro*, the phage cocktail disrupted *P. aeruginosa* biofilm dispersals more effectively when combined with antibiotics (31). The efficacy of both meropenem and phages against *P. aeruginosa* was enhanced when used together (30). Building upon this foundation, the present study focuses on dissecting cell-type-specific mechanisms underlying immunophage synergy during lung infection, particularly the relative contributions of neutrophils and alveolar macrophages to therapeutic efficacy. Here, we aim to further deepen our mechanistic understanding of the therapeutic potential of this phage cocktail by examining its interaction with the innate immune response against *P. aeruginosa*. To achieve this, we employed *in vitro* opsonophagocytic killing assays (OPKA) alongside a murine model of *P. aeruginosa* respiratory infection. Our *in vitro* results indicate a synergistic interaction between phages and phagocytes. Specifically, when bacterial burden surpassed a certain threshold, phage monotherapy alone was insufficient, and neutrophils were unable to effectively clear the infection on their own. *In vivo*, immunophage synergy occurred for neutrophils, but not for alveolar macrophages. These results underscore the importance of neutrophil-phage synergy in enhancing bacterial clearance, emphasizing neutrophils as key contributors to the therapeutic efficacy of the phage cocktail.

## 2 Materials and methods

### 2.1 Animals and animal ethics

Animal studies were approved by the institutional and local governmental authorities at the Charité – Universitätsmedizin Berlin and Landesamt für Gesundheit und Soziales (LaGeSo) Berlin. Animal housing and experimental procedures were performed in compliance with the Federation of European Laboratory Animal Science Associations (FELASA) guidelines and recommendations for the care and use of laboratory animals. Female 8 to 10-week old C57BL/6J WT mice (Janvier Labs, Le Genest-Saint-Isle, France) were housed in randomly assigned groups under specific pathogen-free conditions in individually ventilated cages with free access to food and water and a 12-hour (h) light/dark cycle. Mice were monitored for body temperature (BAT-12 Microprobe, Physitemp, Clifton, NJ, USA), body weight and general condition every 12 h post-depletion and for additional clinical signs of *P. aeruginosa*-induced pneumonia at 12 and 24 h post-infection (hpi). A pre-defined score sheet and a clinical disease score adapted from Wienhold et al. (32) (see **Supplementary Table S1**) based on specific murine pneumonia indicators were used.

#### 2.1.1 Depletion of alveolar macrophages and neutrophils in mice

For the depletion of alveolar macrophages (AlvM), mice were anaesthetized by intraperitoneal (i. p.) injection of ketamine-xylazine (93.75 mg/kg ketamine, 15 mg/kg xylazine), followed by intubation using a laryngoscope (HSE Laryngoscope for mice, Part. No: 73-5072, Hugo Sachs Elektronik Havard Apparatus GmbH, Germany). Clodronate-liposomes (batch no. C04E0222; Liposoma BV, Amsterdam, Netherlands) or PBS-filled liposomes (batch no. P06L0222; Liposoma BV, Amsterdam, Netherlands) as control were then administered intratracheal (i. t.) in a final volume of 80 µL. Mice displayed transient mild loss of body weight and were allowed to recover their starting weight for three days prior infection. For the depletion of polymorphonuclear cells (PMNs) mice were injected i. p. with 200 µg/200 µL of InVivoPlus anti-mouse Ly6G antibody (clone 1A8; Cat# BP0075-1, Bio X Cell, Lebanon, USA) or InVivoPlus rat IgG2a, κ isotype control (clone 2A3; Cat# BP0089, Bio X Cell, Lebanon, USA) pre-pared in sterile 1 × DPBS one day prior infection. Cell depletion rates were assessed via flow cytometry analysis. Mice displayed no adverse effects to depletion and were infected one day post treatment.

Flow cytometric analysis confirmed successful depletion (**Supplementary Figure S3**). Depletion rates in percent were determined by calculating the ratio of cell counts in individual depleted animals to the mean cell counts of the corresponding undepleted control group multiplied by 100%. Control undepleted mice, including i. t. PBS-filled liposomes and i. p. isotype control exhibited no significant differences and were thus grouped together as one common undepleted control group.

#### 2.1.2 Intranasal *P. aeruginosa* infection and phage cocktail treatment

Immune cell depleted (2.1.1) and control mice were anaesthetized by i. p. injection of ketamine-xylazine (93.75 mg/kg ketamine, 15 mg/kg xylazine). Mice were monitored until the loss of the pedal reflex and infected intranasally (i. n.) with 20 µL bacterial suspension containing 5 × 10^6^ CFUs of PAO1 in sterile 1 × DPBS. Control (sham-infected) mice received 20 μL 1 × DPBS, while corneas were protected under anesthesia by Thilo-Tears Gel (Alcon-Deutschland GmbH, Aschaffenburg, Germany). Mice were allowed to recover from anesthesia under an infrared heat source. At 2 hpi, mice were treated i. p. once with 100 µL of the active or UV-inactivated (treatment control group) anti-*P. aeruginosa* phage cocktail (approx. 5 × 10^7^ PFU/injection per phage). A non-lethal inoculum enabling analysis of acute responses was used, and phages were administered 2 hpi as described previously (18).

#### 2.1.3 Dissection and organ sampling

At 24 hpi, mice were euthanized with ketamine (200 mg/kg body weight) and xylazine (20 mg/kg body weight) and blood, bronchoalveolar lavage (BAL), lung and spleen samples were collected for further analysis. After final exsanguination via the vena cava, the lungs were lavaged twice with 0.8 mL of 1 × DPBS protease inhibitor (PI) solution (complete™, Mini Protease Inhibitor Cocktail; Roche, Basel, Switzerland). Lungs were perfused through the right ventricle with 10 mL 1 × DPBS. The right lungs lobes were homogenized in 1 mL 1 × DPBS PI solution using gentleMACS™ M tubes (Miltenyi Biotec, Bergisch Gladbach, Germany) and the left lung lobe minced and digested for cell isolation. Spleens were removed and homogenized in 1 mL 1 × DPBS PI solution using gentleMACS™ M tubes. Blood was collected in EDTA tubes (Sarstedt, Nümbrecht, Germany) and centrifuged at 1500 x g for 10 min at 4°C. Plasma was frozen in liquid nitrogen and stored at -80°C until further analysis. Bacterial load was measured in BAL, homogenized organs (lungs, spleen) and whole blood as described in 2.2.1. Determination of phage load was done using supernatants of centrifuged homogenized organs (1,250 × g, 10 min, 4°C) and whole blood as described in 2.2.2.

#### 2.1.4 Single-cell suspension preparation and flow cytometry

Innate immune cells (leukocytes) in BAL and lungs were analyzed by flow cytometry as described previously (33). To obtain single-cell suspensions, minced lungs were first digested in RPMI media containing collagenase II (Biochrome, Berlin, Germany) and DNAse I (PanReac AppliChem, Darmstadt, Germany) and incubated for 30 min at 37 °C, followed by filtration using a 70 µm cell strainer (BD, Heidelberg, Germany). The suspension was centrifuged (470 × g, 5 min, 4 °C) and the cell pellet resuspended in 2 mL 1 × red blood cell lysis buffer (Santa Cruz Biotechnology, Dallas, USA) for 2 min. Cells were re-washed and centrifuged as described above, cell pellets resuspended in PBS/0.1% BSA buffer and kept on ice. Next, BAL and lung cells were blocked with anti-CD16/CD32 (2.4G2; Cat# 553142, RRID: AB_394657, BD, Heidelberg, Germany) for 3 min and stained with anti-CD45 (30-F11; Cat# 553080, RRID: AB_394610, BD, Heidelberg, Germany), anti-CD11c (N418; Cat# 117310, RRID: AB_313779, BioLegend, San Diego, USA), anti-CD11b (M1/70; Cat# 25-0112-82, RRID: AB_469588, eBioscience, Frankfurt, Germany), anti-F4/80 (BM8; Cat# 12-4801-80, RRID: AB_465922, eBioscience, Frankfurt, Germany), anti-Ly6G (1A8; Cat# 551459, RRID: AB_394206, BD, Heidelberg, Germany), anti-Ly6G (HK.1.4; Cat# 128033, RRID: AB_2562351, BioLegend, San Diego, USA), anti-MHCII (M6/114.15.2; Cat# 12-5322-81, RRID: AB_465930, eBioscience, Frankfurt, Germany), or anti-Siglec F (E50-2440; Cat# 565934, RRID: AB_2739398, BD, Heidelberg, Germany) monoclonal antibodies (mAbs) for 15–20 min. Cells were washed, resuspended, and measured and analyzed on a BD FACSCanto™ II (BD, Heidelberg, Germany). For calculation of total cell numbers CountBright Absolute Counting Beads (Thermo Fisher Scientific, Waltham, USA) were used. For the gating strategy refer to **Supplementary Figure S1** (**Supplementary Materials**).

#### 2.1.5 Total protein in BALF

To quantify lung barrier damage the total protein levels in the murine BAL fluid (BALF) were quantified using the colorimetric DC Protein Assay (Bio-Rad Laboratories, Inc., Hercules, CA, USA) and analyzed via SkanIt Software for Microplate Readers RE, ver 1.0.1.4. Standard curves were created using bovine serum albumin according to the manufacturer’s recommendations.

#### 2.1.6 Cytokine and chemokine quantification

Inflammatory cytokines and chemokines in mouse plasma and BALF samples were measured using the LEGENDplexTM Mouse Inflammation Panel (13-plex with V-bottom plates; BioLegend, San Diego, CA, USA) according to the manufacturer’s recommendations and the provided BioLegend software.

### 2.2 Bacterial strains, bacteriophages, and cell culture

The *P. aeruginosa* strain PAO1 was used for the experiments. Bacterial cryostocks were streaked onto Columbia agar plates containing 5% sheep blood (BD, Heidelberg, Germany) and incubated overnight at 37°C with 5% CO_2_. Bacterial cultures were then prepared from single colonies (OD_600_ = 0.05–0.08) in trypticase soybean broth (TSB, Caso Bouillon, Carl Roth, Karlsruhe, Germany) and incubated at 37°C with orbital shaking at 220 rpm until the early logarithmic phase (OD_600_ = 0.2–0.3) was reached. After centrifugation [1,250 × g for 10 min at room temperature (RT)], the pellet was resuspended in sterile 1 × Dulbecco’s phosphate-buffered saline (DPBS without magnesium and calcium, Thermo Fisher Scientific, Waltham, MA, USA) and adjusted to the desired inoculation dose for *in vivo* (5 × 10^6^ CFU/20 µL) or *in vitro* (indicated multiplicity of infection (MOI) = 0.5 – 50) experiments.

The anti-*P. aeruginosa* phage cocktail used *in vivo* consisted of two *Pseudomonas* phages, DSM 19872 (JG005) (34) and DSM 22045 (JG024) (35), provided by the DSMZ (Braunschweig, Germany) and ITEM Fraunhofer (Braunschweig, Germany) (**Table 1**). These phages were originally selected for their broad lytic activity against diverse *P. aeruginosa* strains within the framework of the Phage4Cure consortium. The individual phage suspensions were highly purified by providing institutions through chromatographic procedures, and endotoxins were removed to low, non-immunogenic levels suitable for preclinical application (29). *In vitro* experiments used a different batch of the same phages mixed with 0.25% human serum albumin (HSA) to increase phage stability. The phage cocktail was prepared by mixing the individual phage suspensions immediately prior to each *in vivo* (approx. 5 × 10^7^ plaque-forming units (PFU)/phage per injection) or *in vitro* (MOI = 0.05) experiment in phage buffer (0.1 M NaCI, 8 mM MgSO4, 50 mM Tris-HCI, pH 7.2–7.5) or 1 × DPBS to ensure accurate phage titers. Phage titers of the original phage suspensions were confirmed with the corresponding indicator strains as reported previously (29) (**Table 1**). The culture conditions of the indicator strains were the same for the analysis strain PAO1, explained above. For each phage in the cocktail, the lytic activity against the bacterial strains used was confirmed *in vitro* via plaque assay. UV-inactivation of the phage cocktail was performed using a UVC 500 Ultraviolet Crosslinker (Hoefer, Inc., San Francisco, CA, USA) for 2 h at 99,900 µJ/cm^2^ and confirmed by the absence of plaque-forming units (PFUs) of control treated mice analyzed via plaque assay (**Supplementary Figure S2**).

**Table 1:** Anti-*Pseudomonas* phage cocktail composition (*in vivo* experiments).

Bacteriophage	Characteristics	Endotoxin level (EU/100 µL injection)	Source
JG005 (DSM 19872) (34)	Class: *Caudoviricetes* Genus: *Pakpunavirus* Morphology: *Myovirus* Indicator strain: F2230 Analysis strain: PAO1 (DSM 22644)	4.30 × 10^1^	DSMZ (Braunschweig, Germany) and Fraunhofer ITEM (Braunschweig, Germany)
JG024 (DSM 22045) (35)	Class: *Caudoviricetes* Genus: *Pbunavirus* Morphology: *Myovirus* Indicator strain: PA14 (DSM 19882) Analysis strain: PAO1 (DSM 22644)	8.08 × 10^-1^	DSMZ (Braunschweig, Germany) and Fraunhofer ITEM (Braunschweig, Germany)

#### 2.2.1 Bacterial burden

Bacterial loads were determined by plating serial dilutions in sterile 1 × DPBS on Columbia agar plates containing 5% sheep blood and overnight incubation (37°C, 5% CO_2_) before calculating the colony-forming units (CFU)/mL tissue sample. The agar plates are not selective but support the growth of *Pseudomonas*. The detection limit was 20 CFU/mL.

#### 2.2.2 Plaque assay

PFUs of tissue samples were determined by serial dilutions (1:10) in phage buffer prior to plaque testing. Bacterial cultures were prepared from single colonies (OD_600_ = 0.05–0.08) and cultured by orbital shaking (220 rpm, 37°C) to early log phase (OD_600_ = 0.2–0.3). Soft agar (4 mL/glass tube) was melted in a heating block (110°C for 10 min) and cooled to 48°C. For the spot test, 100 µL of bacterial suspension was added to the liquid soft agar (top), gently mixed and plated onto agar plates (bottom). Sample dilutions (4 µL/spot) were then plated in triplicate on the plates. The agar plates were incubated overnight (37°C, 5% CO_2_) before calculating the phage load per tissue sample. The detection limit of the triplicate spotted was 83 PFU/mL.

#### 2.2.3 Phage-bacteria visualization by transmission electron microscopy analysis

To preserve the interaction of phages on bacteria in a near-native state for transmission electron microscopy (TEM) analysis, high pressure freezing and freeze substitution (HPF/FS) was used. The TEM was kindly done by the Core Facility Electron Microscopy of Charité Berlin. Samples were prepared for HPF/FS, by incubating the bacterial suspension prepared with single colonies in DPBS (McFarland = 0.5) with the undiluted phage cocktail for 20 min. Afterwards, 7 µL of bacteria/phage solution was placed in the center of a 4 mm polyester membrane disc (0.4 µm pore size) that was placed within the 200 µm indented side of a type A aluminium carrier (Ø 6.0 x 0.5 mm, Nr. 16770126, Leica, Wetzlar, Germany). A type B aluminium carrier (Ø 6.0 x 0.5 mm, Nr. 1677012, Leica, Wetzlar, Germany) was placed on top, flat side down, to form a sandwich. Assembled sandwiches were frozen with liquid nitrogen (-290°C) under 2100 bar using an EM HPM 100 High-Pressure Freezer (Leica, Wetzlar, Germany).

Directly after HPF, frozen carriers containing the disc were transferred in liquid nitrogen into a precooled chamber (-90°C) of an automated freeze substitution (AFS) system (AFS2, Leica, Wetzlar, Germany). Within the chamber, samples were removed from the nitrogen and transferred into precooled (-90°C) Eppendorf tubes containing acetone + 0.2% uranyl acetate (UAc) + 0.2% glutaraldehyde (GA) + 0.5% osmium tetroxide (OsO4) + 1% methanol + 5% water. Samples were gradually substituted over the course of ~22 h from -90°C to 0°C (2 h at 90°C, 2 h at -50°C, 5°C increase/h).

Following FS, samples were processed for embedding and then flat-embedded in epoxy Epon resin. 70 nm ultrathin sections were cut using an ultramicrotome (Leica, Wetzlar, Germany) equipped with a 45° diamond knife (Diatome, Nidau, Switzerland). Sections were collected on pioloform-coated copper grids and stained with lead citrate according to Reynolds (36). Samples were imaged using a Zeiss Leo 906 electron microscope at 80 kV acceleration voltage, equipped with a slow scan 2K CCD camera (TRS, Moorenweis, Germany).

#### 2.2.4 Cell culture and differentiation of HL-60 cells

HL-60 cells, a human promyelocytic leukemic cell line, were purchased from the DSMZ-German Collection of Microorganisms and Cell Cultures GmbH (ACC3, Lot-nr.: 23, Charge-nr.: 2; Leibniz Institute, Braunschweig, Germany) and cultured according to the manufacturer’s recommendations. In brief, cells were recovered after cryogenic storage in RPMI 1640 medium (Thermo Fisher Scientific, Waltham, MA, USA), 20% heat-inactivated (56°C, 30 min) fetal calf serum (FCS, Gibco™ Thermo Fisher Scientific, Waltham, MA, USA), 1% Penicillin-Streptomycin (Thermo Fisher Scientific, Waltham, MA, USA) and 1% L-glutamine (Thermo Fisher Scientific, Waltham, MA, USA). Cells were cultured at 37°C with 5% CO_2_ for two weeks. After this, cells were maintained for additional 4–6 weeks in fresh media with reduced FCS (10%). For the differentiation into neutrophil-like cells (PMN-dHL-60), cells were challenged at 4 × 10^5^ cells/mL with medium containing 0.9% (v/v) dimethylformamide (DMF; Sigma Aldrich, Burlington, MA, USA) for 5–6 days prior to use. For the differentiation into macrophage-like cells (Mϕ-dHL-60), cells were seeded at 4 × 10^5^ cells/mL in a flat-bottom plate (Sarstedt, Nümbrecht, Germany) with medium containing 50 nM Phorbol-12-myristate-13-acetate (PMA; EMD Millipore Corp., Burlington MA; UAS) for 2 days prior to use.

#### 2.2.5 Opsonophagocytic killing assay

For the OPKA serial dilutions (1:4) of anti-*P. aeruginosa* serum (Sigma Aldrich, Burlington, MA, USA) were prepared in a 96-well round-bottom microtiter plate (Sarstedt, Nümbrecht, Germany) with plate buffer (Hank’s Balanced Salt Solution (HBSS) with 0.5 mM MgSO_4_, 0.9 mM CaCl_2_ and 0.1% gelatin; ThermoFisher Scientific Inc., Burlington, MA, USA). For the first incubation step, in which the specific antibodies of the serum will capture the bacteria, a frozen aliquot of PAO1 was thawed in plate buffer to a final concentration of 5 × 10^4^ CFU/mL, the bacterial suspension was added to all wells and the plates were incubated for 15 min at 37°C with shaking at 200 rpm in a horizontal shaker. In a second incubation step to initiate the phagocytosis process, baby rabbit complement (tissue culture grade, CL3441-S; Cedarlane Labs, Burlington, Canada or ref. nr. C12CA.1; Bio-Rad Laboratories, Inc., Hercules, CA, USA), the dHL-60 cells (4 × 10^5^ cells/well) and the phage cocktail in different concentrations (MOI = 1–50) were added. The required volume of PMN-dHL-60 cells was centrifuged at 160 × g for 5 min at RT, the supernatant was discarded and the cell pellet, after an additional washing step resuspended in cell buffer (HBSS with 0.5 mM MgSO_4_, 0.9 mM CaCl_2_,1% gelatin and 5% heat-inactivated FCS) to a final concentration of 1,33 × 10^7^ cells/mL. In case of the Mϕ-dHL-60 cells, the bacterial-antibody suspension from step one in addition to the complement and the phage cocktail were added to the adherent differentiated cells. The phage cocktail was assembled as described above. Each plate also contained controls without serum, complement, immune cells, or phage cocktail; buffer was added in appropriate amounts, respectively. The plates were incubated for 90 min at 37°C with shaking at 200 rpm, a time range identified in preliminary experiments to cover both phagocytosis and phage-mediate bacterial lysis to occur. For calculating the bacterial loads triplicates per sample were plated on blood-agar plates (5% sheep blood) and incubated overnight at 37°C and 5% CO_2_ as described above. Relative CFU counts (%) were calculated by normalization against phagocytosis rate of dHL-60 cells without serum or phages.

### 2.3 Statistical analysis

Data analysis was performed with Prism10 (San Diego, CA, USA). For grouped analyses, ordinary one-way or two-way analysis of variance (ANOVA) with Tukey’s multiple comparison test was used. This approach is considered sufficiently robust to moderate deviations from normality, taking into account the data type and sample size. Results were considered significant when P<0.05. Significance levels are indicated in figures; group sample sizes are indicated in figure legends. CFU and PFU data were logarithmized [Y = log(CFU/PFU+1)] as indicated. Detection limits are given in the figures.

## 3 Results

We previously showed that repeated phage cocktail treatment induces minimal adverse immune response in mice (29), and that the same phage cocktail acts synergistically with the broad-spectrum antibiotic meropenem against *Pseudomonas*-ventilator associated pneumonia (30). In the present study, we investigated the role of host innate immunity for the phage cocktail’s therapeutic efficacy.

### 3.1 Phagocytosis by innate immune cells and phages act synergistically *in vitro*

Neutrophils are the primary effector cells targeting extracellular pathogens like *P. aeruginosa*, which can evade phagocytic killing through several resistance mechanisms. To investigate the interaction between phages and innate immunity, we performed *in vitro* opsonophagocytic killing assays (OPKA) supplemented by a *Pseudomonas*-specific phage cocktail. To this end, we used both, neutrophil-like cells (PMN-dHL-60) and macrophage-like cells (Mϕ-dHL-60) differentiated from human HL-60 cells.

**Figure 1:**
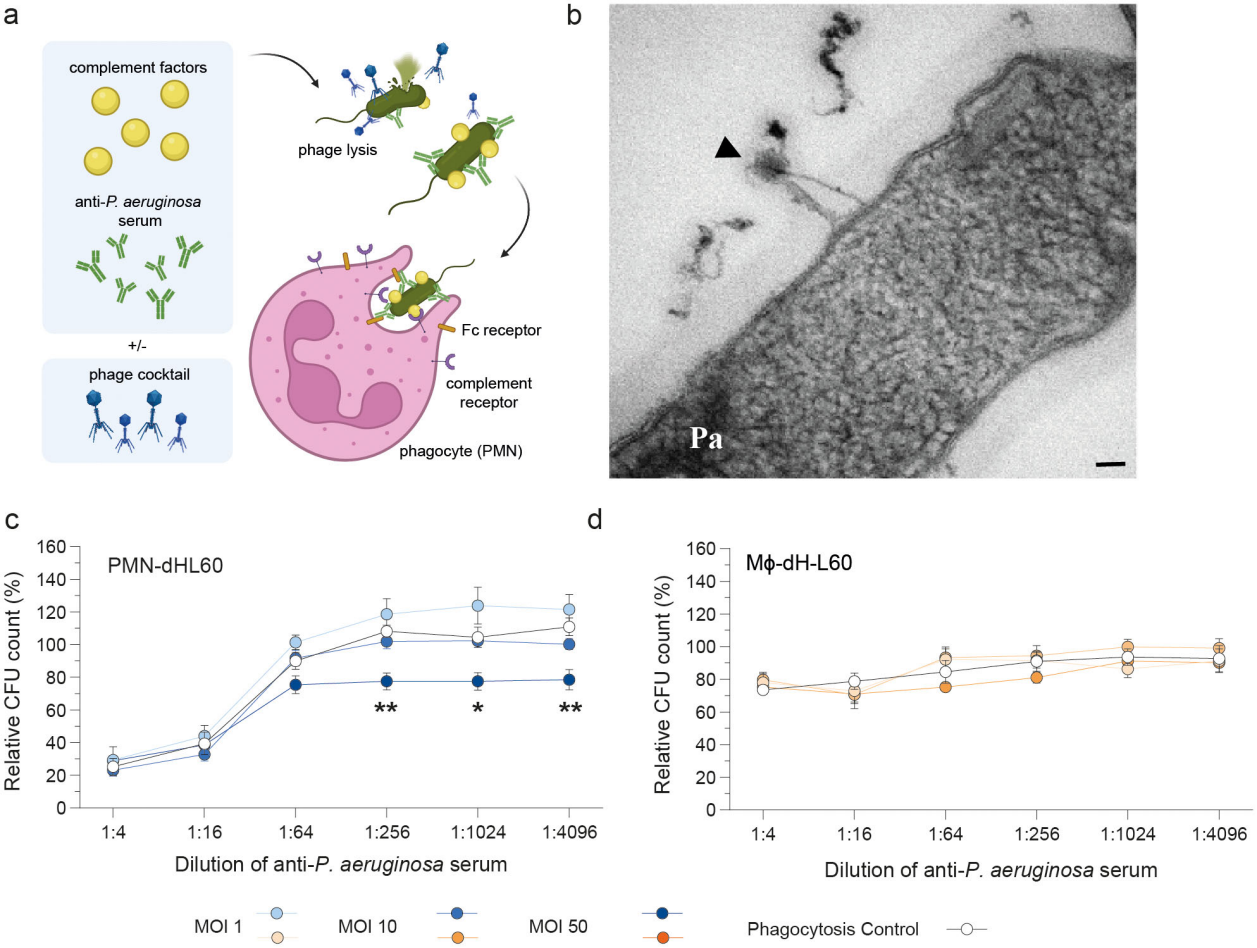
Efficacy of opsonophagocytosis is strengthened by phages *in vitro*. **(a)** Graphical abstract created with BioRender.com. The *in vitro* opsonophagocytic killing assay (OPKA) was used for the determination of the phagocytosis rate of differentiated neutrophil-like cells (PMN-dHL-60) or macrophage-like cells (Mϕ-dHL-60). *P. aeruginosa* (PAO1) were incubated with serial dilutions of anti-*Pseudomonas* specific antibodies (serum) for 15 min (37 °C, 200 rpm) followed by the addition of baby rabbit complement, dHL-60 cells and the *Pseudomonas*-specific phage cocktail in indicated concentrations. **(b)** Transmission electron microscopy visualization shows phage (JG005/JG024; shown by black arrowhead) binding to PAO1 bacteria. Scale bar 50 nm. **(c, d)** Relative bacterial load (CFU, colony-forming unit) of the OPKA was quantified after 90 min incubation. Samples with dHL-60, serum and complement factors, but no phages serve as (opsono)phagocytosis control. Data determined by 2-way ANOVA with Dunnett´s multiple comparisons test: *p < 0.05; **p < 0.01; n = 4-5. MOI, multiplicity of infection. Pa, *P. aeruginosa* strain PAO1.

Opsonophagocytosis was initiated by pre-incubating *P. aeruginosa* with anti-*Pseudomonas* serum, containing pathogen-specific antibodies (**Figure 1a**). After opsonization, differentiated HL-60 (dHL-60) cells, complement factors and varying multiplicities of infection (MOIs) of the phage cocktail were added. Bacterial survival [relative CFU counts (%)] was quantified after a second incubation period. Phages JG005 and JG024 in the phage cocktail target and lyse *P. aeruginosa* strain PAO1 used in this study (**Figure 1b**) (29–31). Using PMN-dHL-60 cells, we observed that addition of phages at MOI 50 significantly reduced bacterial load at serum dilutions over 1:256, where opsonophagocytic function of the cells was otherwise impaired (**Figure 1c**). The phage-mediated reduction reached approximately 30% compared to the phagocytosis-only control (PMN-dHL-60 without phages). In contrast, a lower phage MOI of 1 or 10 did not achieve comparable bacterial reduction.

In contrast, HL-60 cells differentiated into macrophage-like phagocytes (Mϕ-dHL-60) showed limited capacity to phagocytose *P. aeruginosa*, even with phage cocktail support (**Figure 1d**). Their phagocytic response to serum and complement was lower than that of PMN-dHL-60 cells, though a slight serum-dependent trend was observed. Only the high-dose phage cocktail (MOI 50) modestly enhanced bacterial clearance (~10%) compared to the phagocytosis-only control (Mϕ-dHL-60 without phages), while lower MOIs had no effect.

### 3.2 Successful *in vivo* neutrophil and alveolar macrophage depletion strategies

**Figure 2:**
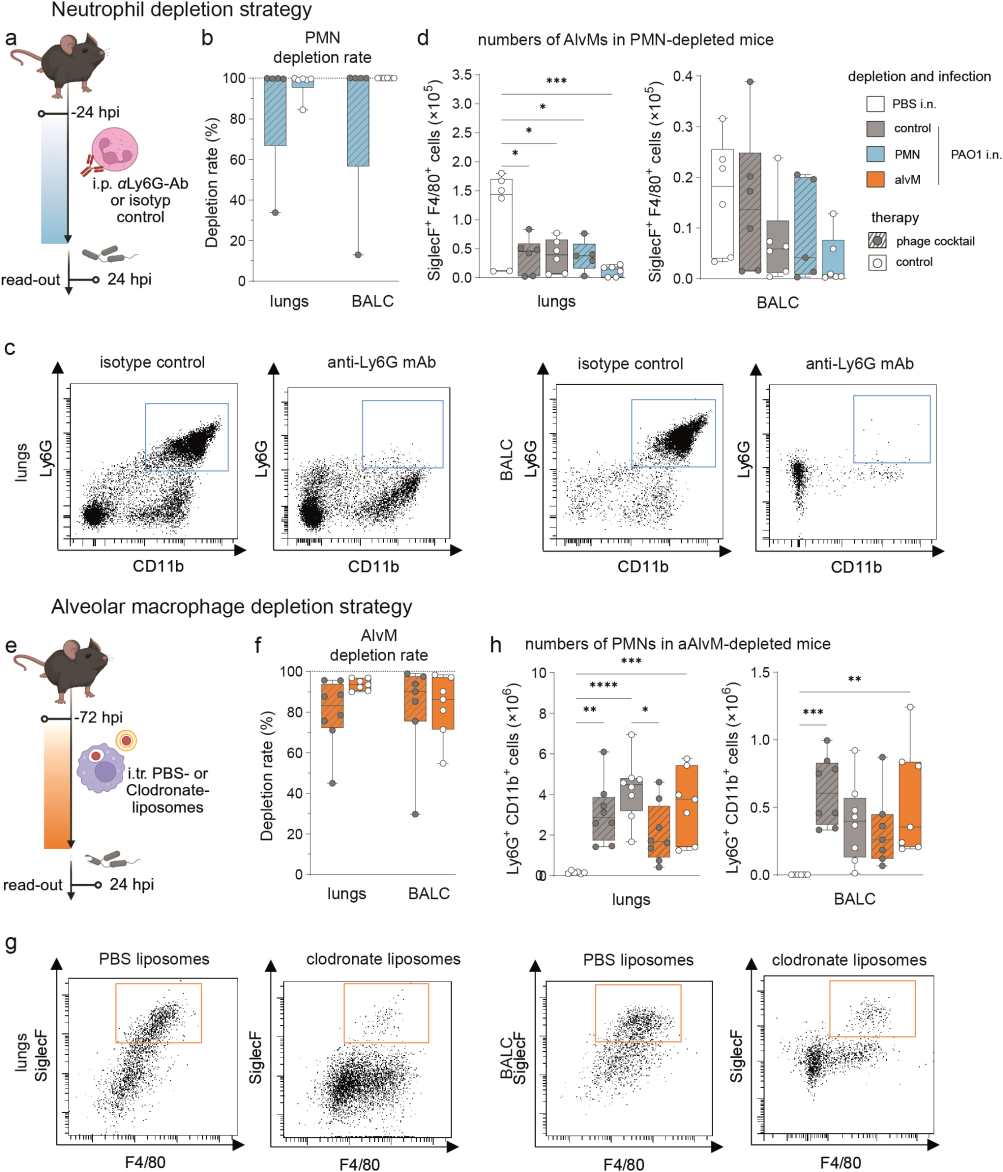
*In vivo* phagocytes depletion strategies. Naive mice (C57BL/6J WT, female, 8–10 weeks; Janvier Labs) were depleted for **(a)** neutrophils (PMNs) via intraperitoneal (i. p.) application of InVivoPlus anti-mouse Ly6G antibody (clone 1A8; Bio X Cell, Lebanon, USA) or InVivoPlus rat IgG2a, κ isotype control (clone 2A3; Bio X Cell, Lebanon, USA) 24 hours or **(e)** alveolar macrophages (AlvM) via intratracheal (i. t.) application of Clodronate- or PBS-filled liposomes as control (Liposoma BV, Amsterdam, Netherlands) 72 hours before infection. Experimental plans created with BioRender.com. **(b, f)** Depletion rates in percent of lungs and BAL cells (BALC). Depletion rates were determined by calculating the ratio of PMNs or AlvM counts in individual depleted animals to the mean PMNs or AlvM count of the corresponding undepleted control group. **(c, g)** Representative dot blots of the flow cytometry analysis at 24 hours post infection (hpi). Numbers of **(d)** AlvMs and **(h)** PMNs determined by flow cytometry. One-way ANOVA, Tukey’s multiple comparisons test. *p < 0,05, **p < 0,01, ***p < 0,001, ****p < 0,0001; depletion control: n = 6 isotype control **(d)** or n = 6 PBS-liposomes (h), sham-infected, control treated mice (white), depletion controls: n = 6 isotype control **(d)** or n = 8 PBS-liposomes **(h)**, PAO1-infected mice with phage treatment (grey, dashed lines), depletion controls: n = 6 isotype control **(d)** or n = 8 PBS-liposomes **(h)**, PAO1-infected, control treated mice (grey), n = 5 mice with PMN cell depletion, PAO1-infection and phage treatment (blue, dashed lines), n = 6 mice with PMN depletion, PAO1-infection and control treatment (blue), n = 8 mice with AlvM depletion, PAO1-infection and phage treatment (orange, dashed lines), n = 7 mice with AlvM cell depletion, PAO1-infection and control treatment (orange). BAL, bronchoalveolar lavage.

Our *in vitro* studies indicated that under certain conditions, such as low antibody titers, phagocytosis efficiency is enhanced by the presence of phages (**Figure 1**). To validate these findings *in vivo*, we employed immune cell depletion strategies in naïve mice, targeting either (i) neutrophils (PMNs) via intraperitoneal injection of the anti-Ly6G antibody clone 1A8, or (ii) alveolar macrophages (AlvMs) through intratracheal administration of clodronate liposomes (**Figure 2**).

To deplete neutrophils, we administered a single intraperitoneal injection of 200 µg anti-Ly6G (clone 1A8) 24 hours prior to infection. This antibody specifically binds Ly6G on neutrophils and mediates their clearance through immune mechanisms (37). Flow cytometry of lung tissue and bronchoalveolar lavage (BAL) fluid at 24 hours post-infection (hpi) confirmed near-complete depletion of neutrophils in both compartments, with the exception of one mouse (**Figures 2a–c**, gating strategy **Supplementary Figure S1**). Moreover, depletion of neutrophils had no major impact on alveolar macrophage numbers (**Figure 2d**).

To deplete alveolar macrophages, we delivered 80 µL of clodronate liposomes intratracheally 72 hours before infection. This strategy exploits the inherent phagocytic activity of macrophages: once internalized, clodronate is released in phagolysosomes, leading to mitochondrial dysfunction and apoptosis (38). At 24 hpi, flow cytometric analysis of lung and BAL leukocytes revealed that alveolar macrophage depletion was maintained at approximately 80–95% (**Figures 2e–g**, gating strategy **Supplementary Figure S1**). Numbers of recruited PMNs in lungs and alveolar spaces were not affected by the depletion of alveolar macrophages at 24 hpi (**Figure 2h**). Further, lung macrophages and dendritic cells (DCs) were not depleted by i. t. clodronate-liposome treatment (**Supplementary Figure S3**).

### 3.3 Antimicrobial phage cocktail efficacy is reduced in the absence of neutrophils, but not in the absence of macrophages

**Figure 3:**
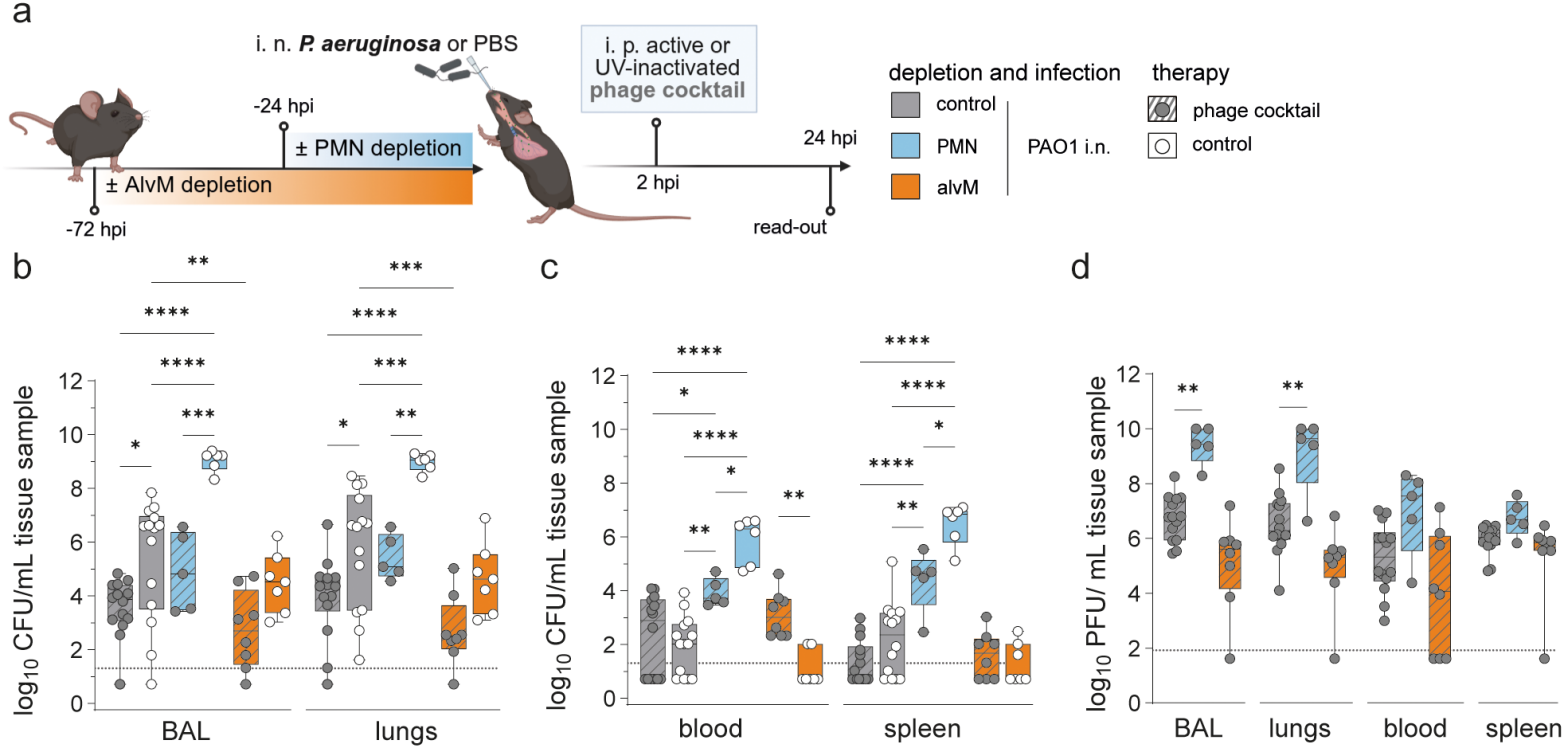
Antimicrobial synergy between phages and innate immune cells. **(a)** Experimental Plan created with BioRender.com. Naive mice (C57BL/6J WT, female, 8–10 weeks; Janvier Labs) were depleted for alveolar macrophages (AlvM) via intratracheal (i. t.) application of Clodronate- or PBS-filled liposomes as control (Liposoma BV, Amsterdam, Netherlands) 72 hours (h) or neutrophils (PMNs) via intraperitoneal (i. p.) application of InVivoPlus anti-mouse Ly6G antibody (clone 1A8; Bio X Cell, Lebanon, USA) or InVivoPlus rat IgG2a, κ isotype control (clone 2A3; Bio X Cell, Lebanon, USA) 24 h before infection. Mice were intranasally (i. n.) infected with PAO1 (5 × 10^6^/20 µL), while sham-infected mice received PBS as control, followed by one intraperitoneal (i. p.) treatment at 2 h post infection (hpi) with a *Pseudomonas*-specific phage cocktail (approx. 5 × 10^7^ PFU/phage per injection). Analysis time point was 24 hpi. Graphs displaying **(b, c)** bacterial loads and **(d)** phage titers at 24 hpi in indicated organs (BAL, homogenized half lungs (right), blood, spleen). Results are shown as box plots depicting median, quartiles, and range. Two-way ANOVA with Tukey’s multiple comparisons test. Significance not shown between PMN and alvM: *p < 0,05, **p < 0,01, ***p < 0,001, ****p < 0,0001; n = 12 control undepleted, sham-infected, control treated mice (white), n = 14 control undepleted, PAO1-infected mice with phage treatment (grey, dashed lines), n = 14 control undepleted, PAO1-infected, control treated mice (grey), n = 5 mice with PMN cell depletion, PAO1-infection and phage treatment (blue, dashed lines), n = 6 mice with PMN depletion, PAO1-infection and control treatment (blue), n = 8 mice with AlvM depletion, PAO1-infection and phage treatment (orange, dashed lines), n = 7 mice with AlvM cell depletion, PAO1-infection and control treatment (orange). Dotted line reflects detection limit. BAL, bronchoalveolar lavage, CFU, colony-forming unit; hpi, hours post infection; PFU, plaque-forming unit.

**Figure 4:**
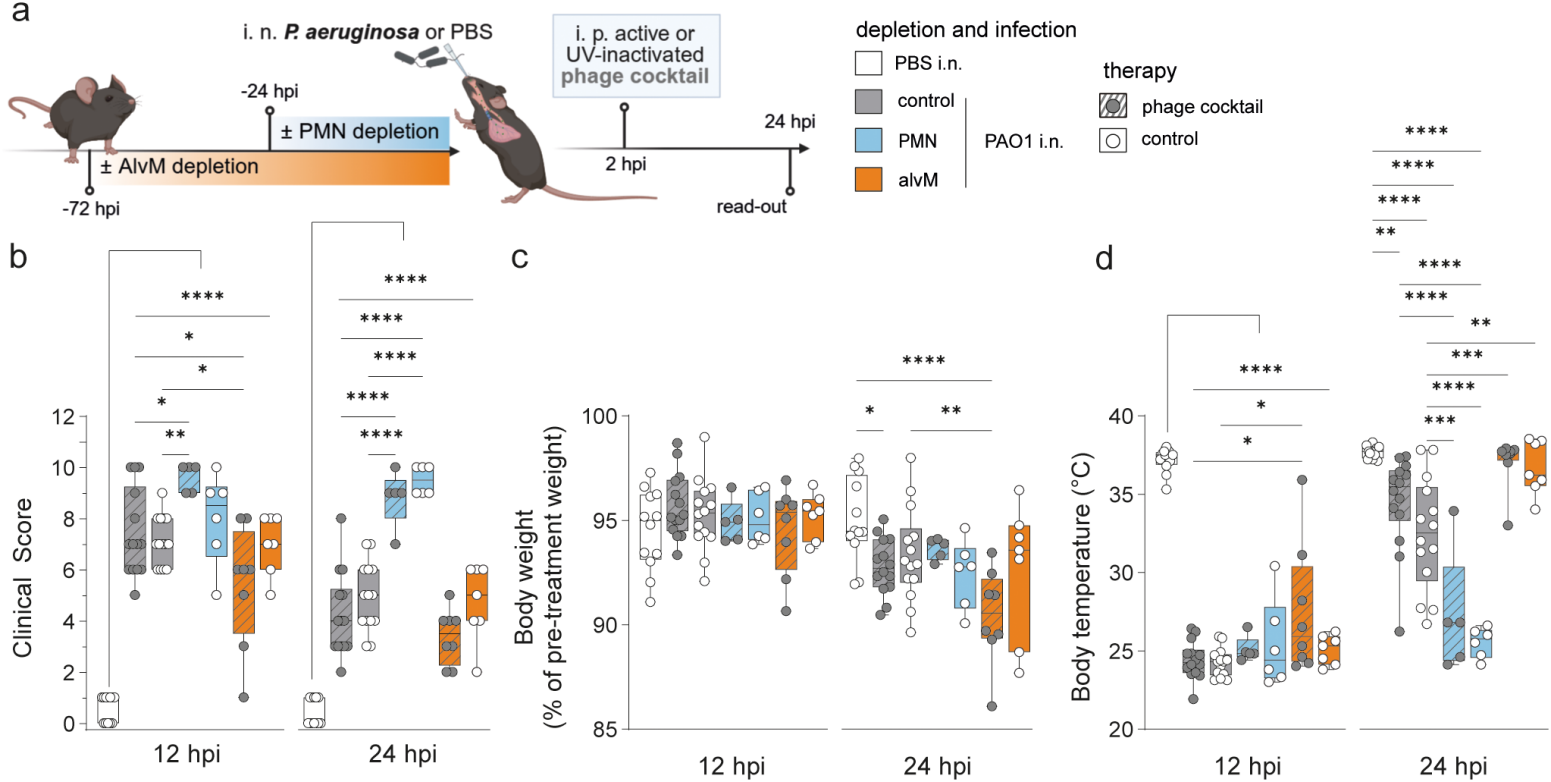
Neutrophils are essential to control *P. aeruginosa* infection in mice, regardless of phage treatment. **(a)** Experimental Plan created with BioRender.com. Naive mice (C57BL/6J WT, female, 8–10 weeks; Janvier Labs) were depleted for alveolar macrophages (AlvM) via intratracheal (i. t.) application of Clodronate- or PBS-filled liposomes as control (Liposoma BV, Amsterdam, Netherlands) 72 hours (h) or neutrophils (PMNs) via intraperitoneal (i. p.) application of InVivoPlus anti-mouse Ly6G antibody (clone 1A8; Bio X Cell, Lebanon, USA) or InVivoPlus rat IgG2a, κ isotype control (clone 2A3; Bio X Cell, Lebanon, USA) 24 h before infection. Mice were intranasally (i. n.) infected with PAO1 (5 × 10^6^/20 µL), while sham-infected mice received PBS as control, followed by one intraperitoneal (i. p.) treatment at 2 h post infection (hpi) with a *Pseudomonas*-specific phage cocktail (approx. 5 × 10^7^ PFU/phage per injection). Analysis time point was 24 hpi. Graphs displaying **(b)** murine clinical disease score (see **Supplementary Table S1**), **(c)** body weight in percent of pre-infection weight and **(d)** body temperature (°C) at indicated time points. Results are shown as box plots depicting median, quartiles, and range. Two-way ANOVA with Tukey’s multiple comparisons test. Significance not shown between PMN and alvM: *p < 0,05, **p < 0,01, ***p < 0,001, ****p < 0,0001; n = 12 control undepleted, sham-infected, control treated mice (white), n = 14 control undepleted, PAO1-infected mice with phage treatment (grey, dashed lines), n = 14 control undepleted, PAO1-infected, control treated mice (grey), n = 5 mice with PMN cell depletion, PAO1-infection and phage treatment (blue, dashed lines), n = 6 mice with PMN depletion, PAO1-infection and control treatment (blue), n = 8 mice with AlvM depletion, PAO1-infection and phage treatment (orange, dashed lines), n = 7 mice with AlvM cell depletion, PAO1-infection and control treatment (orange). BAL, bronchoalveolar lavage.

**Figure 5:**
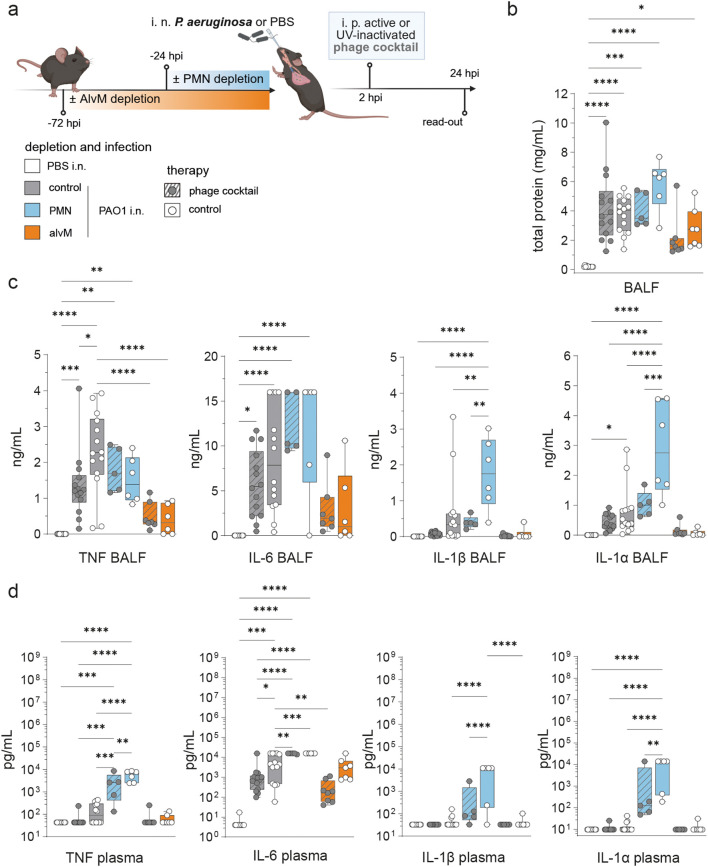
Immune cell depletion prior to *Pseudomonas* infection significantly alters inflammatory cytokine levels **(a)** Experimental Plan created with BioRender.com. Naive mice (C57BL/6J WT, female, 8–10 weeks; Janvier Labs) were depleted for alveolar macrophages (AlvM) via intratracheal (i. t.) application of Clodronate- or PBS-filled liposomes as control (Liposoma BV, Amsterdam, Netherlands) 72 hours (h) or neutrophils (PMNs) via intraperitoneal (i. p.) application of InVivoPlus anti-mouse Ly6G antibody (clone 1A8; Bio X Cell, Lebanon, USA) or InVivoPlus rat IgG2a, κ isotype control (clone 2A3; Bio X Cell, Lebanon, USA) 24 h before infection. Mice were intranasally (i. n.) infected with PAO1 (5 × 10^6^/20 µL), while sham-infected mice received PBS as control, followed by one intraperitoneal (i. p.) treatment at 2 h post infection (hpi) with a *Pseudomonas*-specific phage cocktail (approx. 5 × 10^7^ PFU/phage per injection). Analysis time point was 24 hpi. Graphs displaying **(b)** total protein levels (mg/mL) in BAL fluid (BALF) and level of the pro-inflammatory cytokines TNF, IL-6, IL-1β and IL-1α in **(c)** BALF (ng/mL) or **(d)** plasma (pg/mL) of *Pseudomonas*-infected mice. Results are shown as box plots depicting median, quartiles, and range, as determined by ordinary one-way ANOVA with Tukey´s multiple comparisons test: *p < 0.05; **p < 0.01; ***p < 0.001; ****p < 0.0001; n = 12 control undepleted, sham-infected, control treated mice (white), n = 14 control undepleted, PAO1-infected mice with phage treatment (grey, dashed lines), n = 14 control undepleted, PAO1-infected, control treated mice (grey), n = 5 mice with PMN cell depletion, PAO1-infection and phage treatment (blue, dashed lines), n = 6 mice with PMN depletion, PAO1-infection and control treatment (blue), n = 8 mice with AlvM depletion, PAO1-infection and phage treatment (orange, dashed lines), n = 7 mice with AlvM cell depletion, PAO1-infection and control treatment (orange). BAL, bronchoalveolar lavage; IL, interleukin; TNF, tumor necrosis factor.

Using the above depletion conditions, we next investigated whether the presence of phagocytes, specifically neutrophils and alveolar macrophages, impacts the efficacy of phage therapy in bacterial clearance *in vivo*. To this end, we intranasally infected control-depleted, PMN-depleted, and AlvM-depleted mice with *P. aeruginosa* strain PAO1, sham-infected mice received PBS, respectively. Two hours post-infection (hpi), mice received a single intraperitoneal (i. p.) injection of the two-phage cocktail (~5 × 10^7^ PFU per phage in 100 μL), either active or UV-inactivated (**Figure 3a**). No significant differences in outcome parameters were observed between the two control depletion strategies (isotype control antibody or PBS liposomes). Consequently, data from both control groups were pooled and are presented as a single summarized control group in all graphical analyses (**Figures 2**–**5**).

Bacterial burden analysis confirmed the efficacy of the phage cocktail in eliminating *P. aeruginosa* (29–31). Phage treatment significantly reduced bacterial loads by approximately 3 log units in the BAL and lungs, and by around 2 log units in the blood and spleen (**Figures 3b, c**). Additionally, our results confirmed the critical role of neutrophils in controlling *P. aeruginosa* infection (10). In neutrophil-depleted mice, CFUs were markedly elevated in both local (BALF, lungs) and systemic (blood, spleen) compartments (**Figures 3b, c**). In contrast, bacterial loads in alvM-depleted mice were only slightly reduced, by trend, compared to control-depleted, infected mice (**Figures 3b, c**), suggesting that alveolar macrophages contribute minimally to *P. aeruginosa* clearance. Phage titers (**Figure 3d**) were higher in all compartments of PMN-depleted mice, consistent with enhanced phage replication in the presence of higher bacterial densities.

Confirming our *in vitro* findings, the synergy between phages and neutrophils was particularly evident at the primary infection and treatment sites (BAL and lungs), where both bacterial loads (**Figure 3b**) and phage titers (**Figure 3d**) were high. Mice possessing neutrophils and receiving phage treatment exhibited significantly lower CFUs compared to mice lacking both neutrophils and phage therapy. Interestingly, mice with only one of the two factors, either neutrophils or phage therapy, showed intermediate CFU levels, falling between groups with two advantageous (with neutrophils and phages) and no advantageous factors (no neutrophils, no phages) (**Figures 3b, c**).

Although alveolar macrophages are phagocytic, their presence did not provide a significant advantage against *P. aeruginosa*. However, there was a trend towards greater efficacy of phage therapy when alveolar macrophages were intact, suggesting a possible, albeit minimal, additional effect (**Figures 3b, c**).

### 3.4 Neutrophil depletion delayed recovery from *Pseudomonas*-infection

Next, we evaluated if the neutrophil and phage therapy mediated changes in bacterial burden affected the physiological response to *Pseudomonas* infection (**Figure 4**). All infected mice, regardless of treatment, exhibited elevated clinical scores compared to the sham-infected control group. Clinical scores peaked at 12 hpi, consistent with the expected endotoxin shock response to *P. aeruginosa* infection (**Figure 4b**). Notably, only the PMN-depleted mice failed to show clinical improvement by 24 hpi. Phage therapy showed a trend toward improved clinical scores, but this effect did not reach significance. Changes in body weight, likely due to altered eating and drinking behavior, became evident from 24 hpi onward (**Figure 4c**). The primary correlate of weight loss was infection with *P. aeruginosa*; neutrophil depletion did not exacerbate this effect, and phage therapy did not significantly alleviate it within the observed time window. Surprisingly, mice depleted of alveolar macrophages using clodronate liposomes and treated with phages experienced the most pronounced weight loss at 24 hpi (**Figure 4c**).

In contrast to weight loss, a drop in body temperature was already noticeable at 12 hpi and was similarly driven by infection (**Figure 4d**). Interestingly, mice lacking alveolar macrophages maintained their body temperature better at 12 hpi and showed greater recovery by 24 hpi. This suggests that alveolar macrophages may play a role in mediating the endotoxin shock response responsible for hypothermia in infected mice. By 24 hpi, the most pronounced differences between groups, based on immune cell depletion and phage treatment, became apparent. Phage therapy led to an overall improvement in clinical indicators, including higher body temperatures, suggesting a faster recovery in treated mice. In contrast, PMN-depleted mice failed to recover from *Pseudomonas*-infection, as indicated by persistently low body temperatures and phage treatment had only limited benefit in this group.

### 3.5 Alveolar macrophages worsen lung injury, while neutrophils and phage therapy reduce inflammation

To further evaluate the divergent role of phagocyte phage synergy, we measured the extent of lung barrier damage through total protein levels in the BALF at 24 hpi (**Figures 5a, b**). Differences between groups were not significant but trends emerged. Again, PMN-depleted mice without phage treatment exhibited the severest phenotype, displaying highest protein concentrations. The presence of either phage therapy or neutrophils alleviates the lung barrier damage, suggesting mild synergy in barrier protection.

Earlier, we found that the presence of alveolar macrophages limited recovery from initial hypothermia, which was alleviated by phage therapy (**Figure 4d**). In line, lung barrier damage was lowest when alveolar macrophages were depleted and mice additionally treated with phages. Thus, the absence of alveolar macrophages was barrier protective and phage therapy by trend further enhanced the protective effect (**Figure 5b**).

We next assessed the inflammatory cytokine response in the context of infection, immune cell depletion, and phage treatment. Local tissue and innate immune cells recognize *Pseudomonas* pathogen-associated molecular patterns (PAMPs), such as lipopolysaccharide (LPS), via pattern recognition receptors (PRRs), triggering the release of pro-inflammatory cytokines. To determine whether phage therapy and phagocyte populations influenced this response synergistically or independently, we quantified TNF, IL-6, IL-1β, and IL-1α in both BALF and plasma (**Figures 5c, d**; **Supplementary Figures S4**, **S5**).

Cytokine profiling confirmed previous findings: neutrophils contribute to synergistic effects with phage therapy, while alveolar macrophages are key mediators of the detrimental endotoxin shock response. Overall, phage treatment, both in immunocompetent and immune-depleted mice, reduced levels of pro-inflammatory cytokines, although the extent varied by cytokine. For instance, PMN depletion resulted in markedly elevated levels of IL-6, IL-1β, and IL-1α at 24 hpi. Notably, while phage therapy had limited effect on IL-6 levels, it significantly reduced IL-1β and IL-1α levels in both BALF and plasma. Particularly in BALF, cytokine analysis revealed that prior depletion of alveolar macrophages led to lower levels of TNF, IL-6, IL-1β, and IL-1α (**Figure 5c**).

These findings further support the conclusion that phages and phagocytes can act synergistically under specific conditions, as initially demonstrated *in vitro* using established neutrophil- and macrophage-like cell lines. To translate these observations to a physiologically relevant context, we employed an *in vivo* mouse model with targeted depletion of either neutrophils or alveolar macrophages. These experiments revealed distinct and contrasting roles for the two phagocyte populations during *P. aeruginosa* respiratory infection, highlighting the complexity of immune–phage interactions *in vivo* and underscore the cell-type specificity of phage support.

## 4 Discussion

The global rise of MDR bacteria like *P. aeruginosa* continues to challenge antibiotic therapies, driving renewed interest in phage therapy as a promising alternative or adjunct. The present study builds up on our previous work which demonstrated that a two-phage cocktail, combined with meropenem, enhanced bacterial clearance and reduced inflammation in a murine lung infection model (30). Here, using *in vitro* and *in vivo* models, we demonstrate that our *Pseudomonas* phage cocktail exhibits highest efficacy in the presence of neutrophils, while the reliance on alveolar macrophages for efficacy is neglectable.

These results fully align with previous studies supporting the concept of *immunophage synergy*. In a murine model of multidrug-resistant *P. aeruginosa* pneumonia Roach et al. demonstrated, that phage therapy was most effective when combined with a functioning immune response, particularly neutrophils (10). Neutrophil-phage cooperation was essential not only for bacterial clearance but also for controlling emerging phage-resistant variants. Similarly, a study using the *P. aeruginosa* phage PA1Ø demonstrated high survival rates (80–100%) only in immunocompetent, but not neutropenic, mice (15). Additionally, they demonstrated markedly enhanced *in vitro* bacterial killing when phages were combined with neutrophils, highlighting the importance of co-activity for therapeutic efficacy (15). Interestingly, Cafora et al. demonstrated that phage cocktails targeting *P. aeruginosa* not only reduced bacterial burden and inflammatory cytokine expression in CFTR-deficient zebrafish, as a model for CF, but also exerted anti-inflammatory effects independent of infection, mediated through TLR recognition of phage capsid proteins and resulting in decreased neutrophil recruitment (8, 9).

In line with our OPKA assays, where anti-*Pseudomonas* serum enhanced phagocytosis, anti-*P. aeruginosa* IgY antibodies similarly boost neutrophil respiratory burst and phagocytosis, thereby accelerating bacterial clearance, especially relevant for managing persistent infections in CF patients (39). Furthermore, antibody-based therapies, such as the bispecific monoclonal antibody Gremubamab targeting both Psl and PcrV, have been shown to enhance neutrophil-mediated killing of *P. aeruginosa* and reduce associated cytotoxicity in patients with bronchiectasis (40).

In our murine respiratory infection model, neutrophil depletion not only reduced phage treatment efficacy but also delayed recovery from *P. aeruginosa*-infection, affirming the pivotal role of neutrophils in infection control regardless of phage presence. Conversely, alveolar macrophages appeared to exacerbate lung barrier dysfunction and inflammatory cytokine release during endotoxin challenge, suggesting a potentially detrimental role in this context. This divergent impact of immune cells emphasizes the complexity of host-pathogen-phage interactions and the necessity to consider specific immune cell functions when designing clinical phage therapies. The modulation of inflammatory cytokines by immune cell depletion prior to infection further supports the complex balance between infection control and immunopathology. This complexity is highlighted by findings that neutrophils, while central to clearing *P. aeruginosa* in healthy individuals, often fail in CF, illustrating how pathogen evasion and host immune dysfunction shape treatment outcomes (13). Moreover, consistent with our findings, neutrophil depletion in a murine model of acute *P. aeruginosa* lung infection has been shown to significantly increase mortality, whereas depletion of alveolar macrophages did not produce such an effect (41).

Although alveolar macrophages play a key role in pathogen clearance and tissue repair, their response to endotoxin can exacerbate lung injury by promoting lung barrier breakdown and excessive cytokine release (42, 43). These effects have also been implicated in ventilator-induced lung injury (VILI), where early macrophage activation plays a role in pathogenesis (44). In CF models, macrophages have been implicated in driving exaggerated cytokine responses following *P. aeruginosa* LPS exposure, suggesting a viable therapeutic target to mitigate chronic inflammation and tissue damage (45). Interestingly, while alveolar macrophages have a dual role, protective in some settings but harmful in others, their influence seems minimal in the context of immunophage synergy, where neutrophils remain the dominant effector. Supporting this, a recent modeling study proposed that alveolar macrophages may even limit phage efficacy by lowering the density of therapeutic phages, and that their depletion could enhance phage bioavailability and consequently the survival in *Pseudomonas*-infected mice (16). However, we could not corroborate these findings, as the phage load in our *in vivo* infection model was unaffected by cell depletion status.

The success of phage therapy is tightly linked to the host’s immune competence, especially neutrophil function. Patients with compromised neutrophil responses may exhibit diminished phage therapy efficacy. Independent of *Pseudomonas-*infections, the increasing diversity of multidrug-resistant pathogens, along with the rising numbers of elderly and immunocompromised individuals, underscore the urgent need for translational research in phage therapy (46–48). Furthermore, the involvement of antibodies in enhancing neutrophil-phage cooperation suggests that humoral immunity also plays a critical role, warranting a more integrated view of immunophage synergy in therapeutic settings.

This study has some limitations. First, we used the laboratory strain *P. aeruginosa* PAO1 in our murine respiratory infection model. While PAO1 is a relevant MDR pathogen, it does not fully reflect the diversity of clinical isolates. Future studies using clinical strains could enhance translational relevance but require suitable murine models and phage specificity testing. Second, our experiments were conducted in female mice to minimize sex-related variability in pneumonia severity (49, 50). Accordingly, potential sex-specific immune responses should be explored in future studies. Finally, our analysis was limited to the acute phase of infection with the analysis time point at 24 hpi, which precludes assessment of recovery, and long-term outcomes.

Our work corroborates the understanding that optimizing phage therapy requires not only selecting effective phage cocktails but also recognizing and leveraging the host’s innate and adaptive immune responses. Future clinical strategies should integrate immune profiling to personalize phage-based treatments, thereby maximizing therapeutic success against MDR bacterial infections.

## Data Availability

The raw data supporting the conclusions of this article will be made available by the authors, without undue reservation.
