# Supplementary material for: Neutrophils, not macrophages, aid phage-mediated control of pulmonary Pseudomonas aeruginosa infection
Source: Front Immunol. 2025 Nov 19;16:1681461. doi: 10.3389/fimmu.2025.1681461 (PMC12672430; doi:10.3389/fimmu.2025.1681461)
Supplement: Supplementary file 1 [file DataSheet1.pdf]

Neutrophils, not Macrophages, Aid Phage-Mediated Control of Pulmonary *Pseudomonas aeruginosa* Infection

Chantal Weissfuss[1,2,*] et al.

Supplementary Material

Supplementary Table S1. Clinical disease score for murine model of *P. aeruginosa* respiratory infection. p. i., post infection.

Symptom	Score
**Body Condition Score** (Ullman-Culleré, *et al.* 1999)	
BC3	0
BC2	1
BC1	2
**Body weight change p. i.**	
<5 %	0
>5 % ≤ 10 %	1
>10 % ≤ 20 %	2
>20 %	3
**Behaviour / Temperature p. i.**	
Curious, awaken / > 35 °C	0
Less active / > 35 °C	1
Self-isolation or ≤ 35 °C	2
Self-isolation and ≤ 35 °C	3
**Fur**	
Shiny	0
Slightly dishelved	1
Moderately dishelved, crusty eyes	2
Fur/eyes highly dirty, piloerection	3
**Breathing**	
Steady, consistent	0
Breathing rate increased	1
Hyper- /hypoventilation, slightly abdominal	2
Labored, moderately abdominal	3
**Total clinical score**	

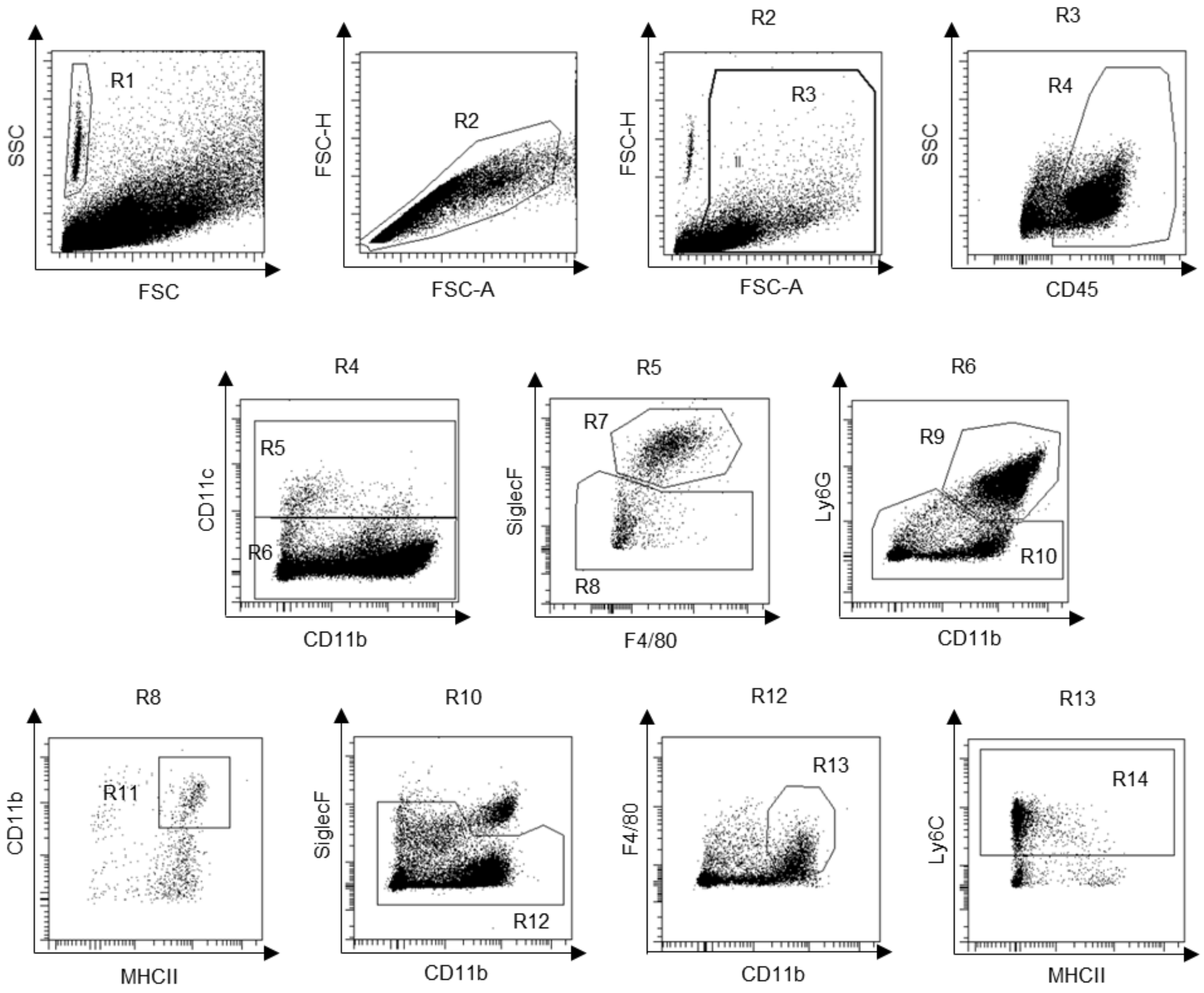

R1 beads
R2 singlets
R3 minus debris
R4 CD45+ cells (leucocytes)
R5 CD45^{+} CD11c^{+} cells
R7 CD45^{+} CD11c^{+} SiglecF^{+} F4/80^{+} (**alvM**)
R8 CD45^{+} CD11c^{+} SiglecF^{-} F4/80^{-} (**DCs**)
R11 CD45^{+} CD11c^{+} CD11b^{+} SiglecF^{-} F4/80^{-} MHCII^{+} (matDCs)
R6 CD45^{+} CD11c^{-} cells
R9 CD45^{+} Ly6G^{-} CD11b^{high} cells (**PMNs**)
R10 CD45^{+} Ly6G^{-} CD11c^{-} cells
R12 CD45^{+} Ly6G^{-} CD11c^{-} SiglecF^{-} cells
R13 CD45^{+} Ly6G^{-} CD11c^{-} SiglecF^{-} CD11b^{+} F4/80^{+} cells (**lung macrophages**)
R14 CD45^{+} Ly6G^{-} CD11c^{-} SiglecF^{-} CD11d^{+} F4/80^{+} Ly6C^{high} cells (Infl.Mo)

Supplementary Figure S1. Exemplary flow cytometric gating strategy for the analysis of innate immune cells. Representative dot plots illustrating the gating strategy of innate immune cells in lungs and BALC. alvM, Alveolar Macrophages; BALC, Bronchoalveolar lavage cells; Infl.Mo, Inflammatory Monocytes; (mat)DCs, (mature) Dendritic Cells; PMNs, Polymorphonuclear Neutrophils.

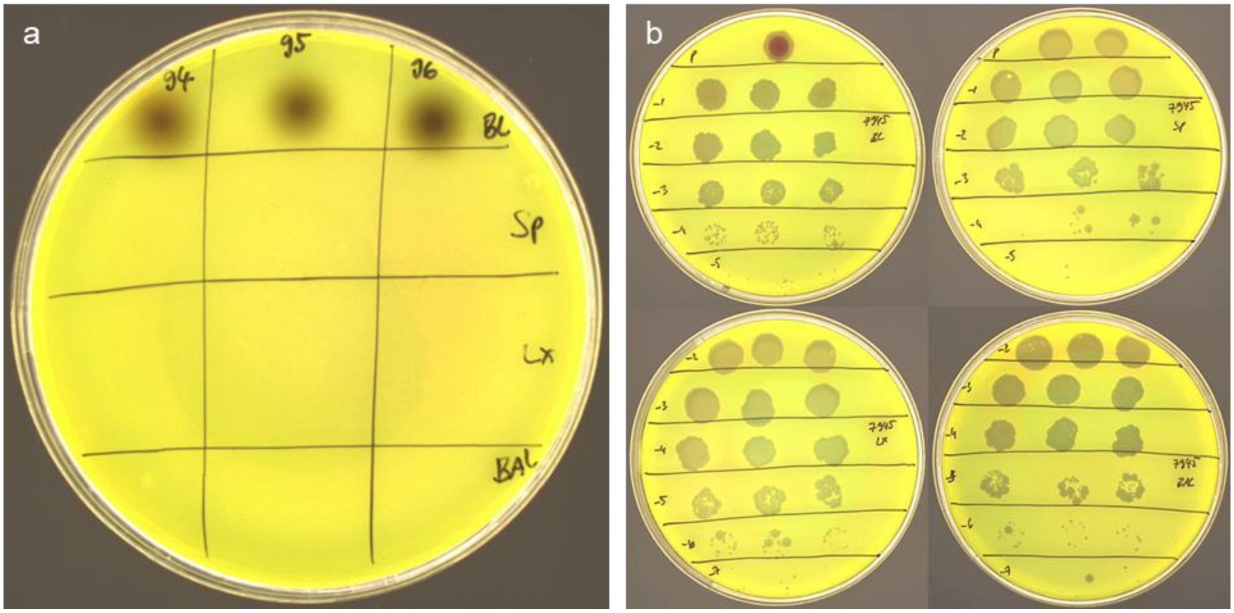

Supplementary Figure S2. Exemplary plaque assay of control treated mice confirming UV-inactivation of the phage cocktail. (**a**) Representative results of the plaque assay of experimental animals receiving the UV-inactivated phage cocktail analyzed at 24 hpi. 4 µL of undiluted organ preparations (BL, blood; BAL, bronchoalveolar lavage; Lx, lung; SP, spleen) were plated and no PFUs were detectable. (**b**) Representative results of the plaque assay of one mouse receiving the active phage cocktail analyzed at 24 hpi. Serial dilutions (p, pure/undiluted to 10^{-9}) of organ preparations were plated in triplicates; clear spots indicate PFUs. PFUs, plaque-forming units.

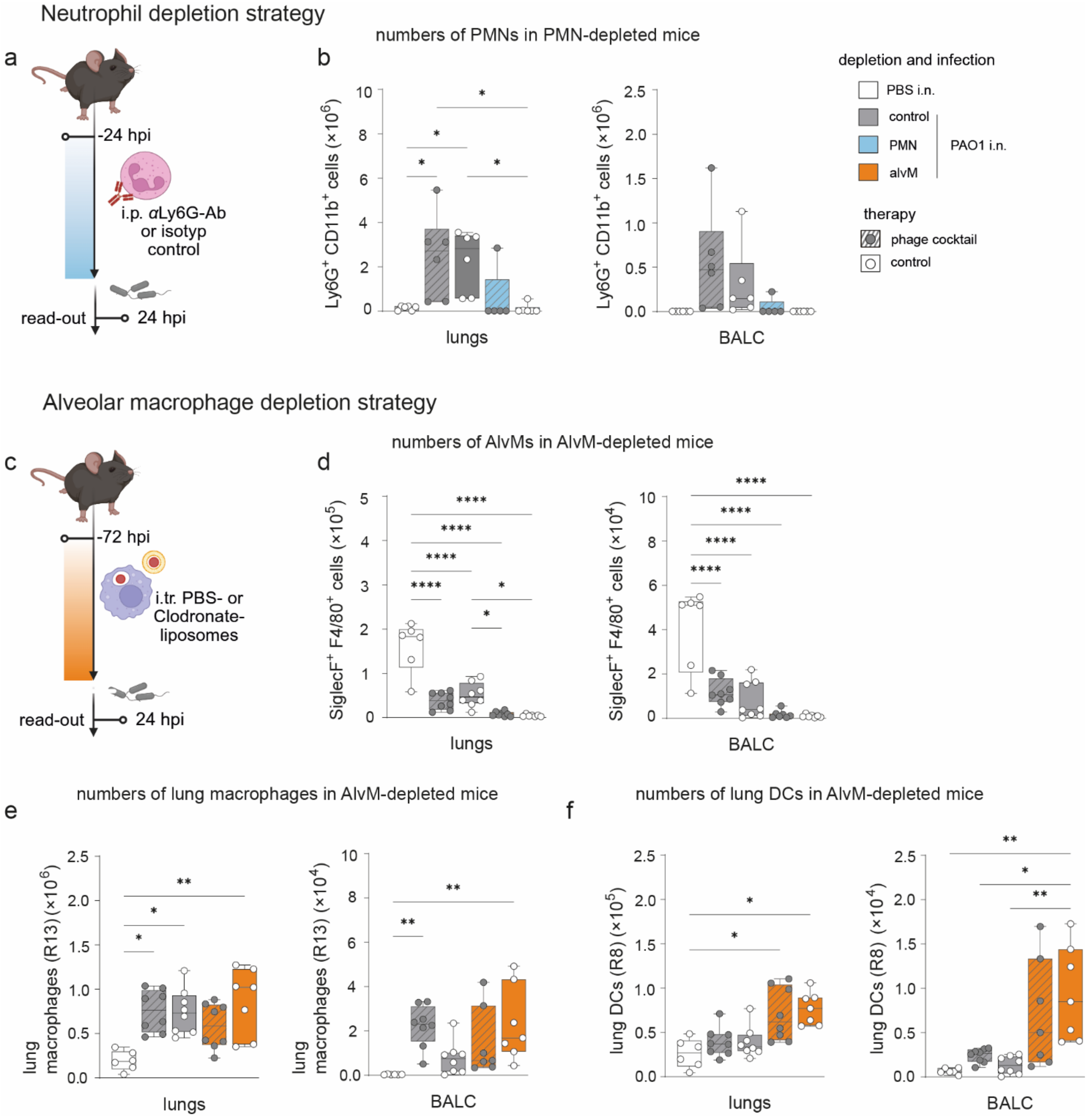

Supplementary Figure S3. Alveolar macrophage depletion via Clodronate-liposomes does not affect interstitial macrophages or DCs. Naive mice (C57BL/6J WT, female, 8–10 weeks; Janvier Labs) were depleted for (**a**) neutrophils (PMNs) via intraperitoneal (i. p.) application of InVivoPlus anti-mouse Ly6G antibody (clone 1A8; Bio X Cell, Lebanon, USA) or InVivoPlus rat IgG2a, κ isotype control (clone 2A3; Bio X Cell, Lebanon, USA) 24 hours or (**c**) alveolar macrophages (AlvM) via intratracheal (i. t.) application of Clodronate- or PBS-filled liposomes as control (Liposoma BV, Amsterdam, Netherlands) 72 hours before infection. Experimental plans created with BioRender.com. Numbers of (**b**) PMNs in PMN-depleted mice as well as numbers of (**d**) AlvM, (**e**) lung macrophages (gate R13, see gating strategy in Supplementary Figure S1) and (**f**) lung DCs (gate R8, see gating strategy in Supplementary Figure S1) in AlvM-depleted mice determined by flow cytometry at 24

hours post infection (hpi). One-way ANOVA, Tukey's multiple comparisons test. *p < 0,05, **p < 0,01, ***p < 0,001, ****p < 0,0001; depletion control: n = 6 isotype control (**b**) or n = 6 PBS-liposomes (**d-f**), sham-infected, control treated mice (white), depletion controls: n = 6 isotype control (**b**) or n = 8 PBS-liposomes (**d-f**), PAO1-infected mice with phage treatment (grey, dashed lines), depletion controls: n = 6 isotype control (**b**) or n = 8 PBS-liposomes (**d-f**), PAO1-infected, control treated mice (grey), n = 5 mice per group with PMN cell depletion, PAO1-infection and phage treatment (blue, dashed lines), n = 6 mice with PMN depletion, PAO1-infection and control treatment (blue), n = 8 mice with AlvM depletion, PAO1-infection and phage treatment (orange, dashed lines), n = 7 mice with AlvM cell depletion, PAO1-infection and control treatment (orange).BAL, bronchoalveolar lavage. DCs, Dendritic cells.

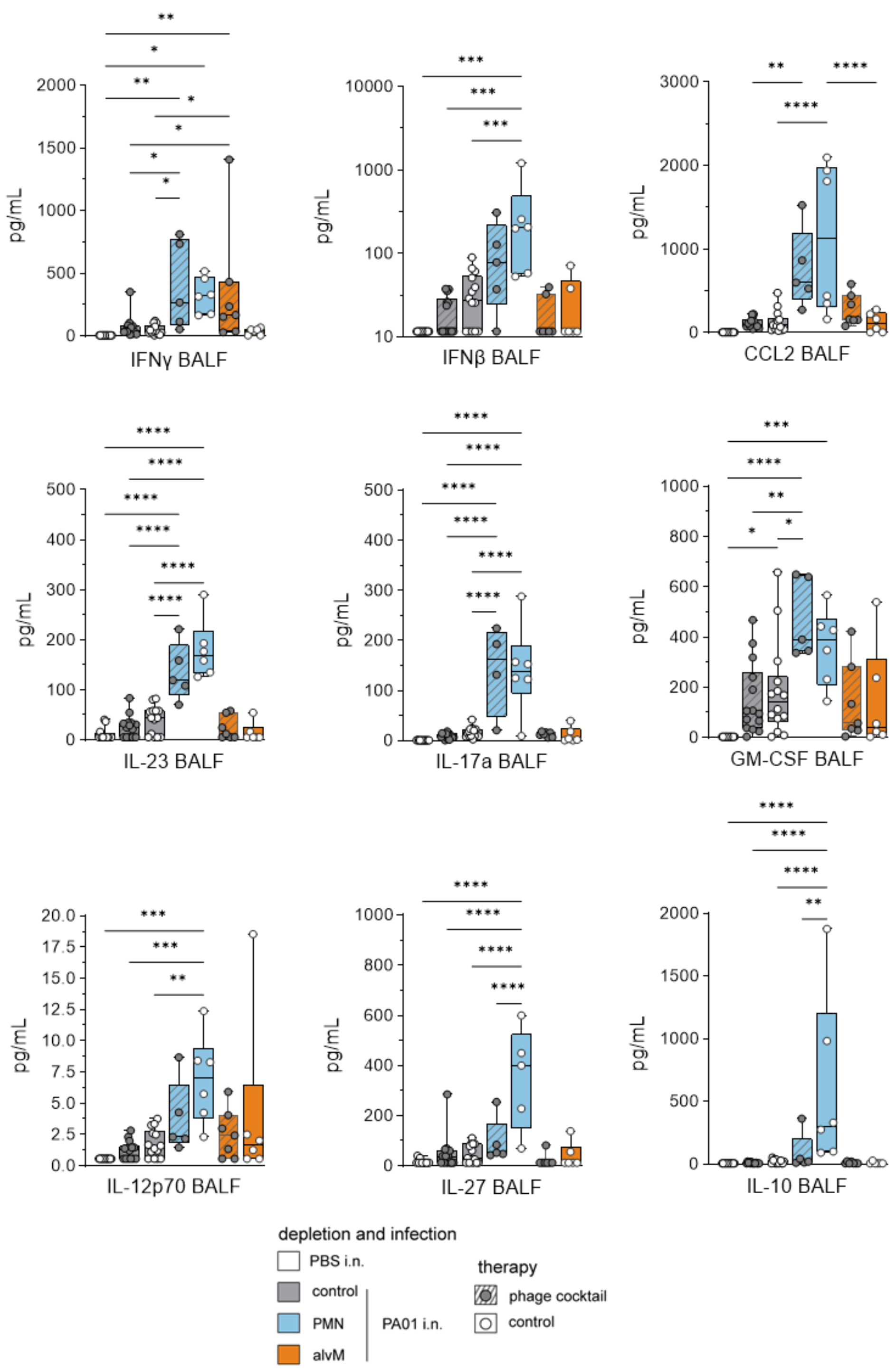

Supplementary Figure S4. Cytokines and Chemokines in BALF of *Pseudomonas*-infected mice. Level of pro-inflammatory cytokines (pg/mL) in BALF of mice analyzed at 24 hpi. Results are shown as box plots depicting median, quartiles, and range, as determined by ordinary one-way ANOVA with Tukey´s multiple comparisons test: *p < 0.05; **p < 0.01; n = 12 control undepleted, sham-infected, control treated mice (white), n = 14 control undepleted, PAO1-infected mice with phage treatment (grey, dashed lines), n = 14 control undepleted, PAO1-infected, control treated mice (grey), n = 5 mice with PMN cell depletion, PAO1-infection and phage treatment (blue, dashed lines), n = 6 mice with

PMN depletion, PAO1-infection and control treatment (blue), n = 8 mice with AlvM depletion, PAO1-infection and phage treatment (orange, dashed lines), n = 7 mice with AlvM cell depletion, PAO1-infection and control treatment (orange). BALF, bronchoalveolar lavage fluid; IL, interleukin; CCL2, chemokine (C-C motif) ligand 2; GM-CSF, Granulocyte-macrophage- colony stimulating factor.

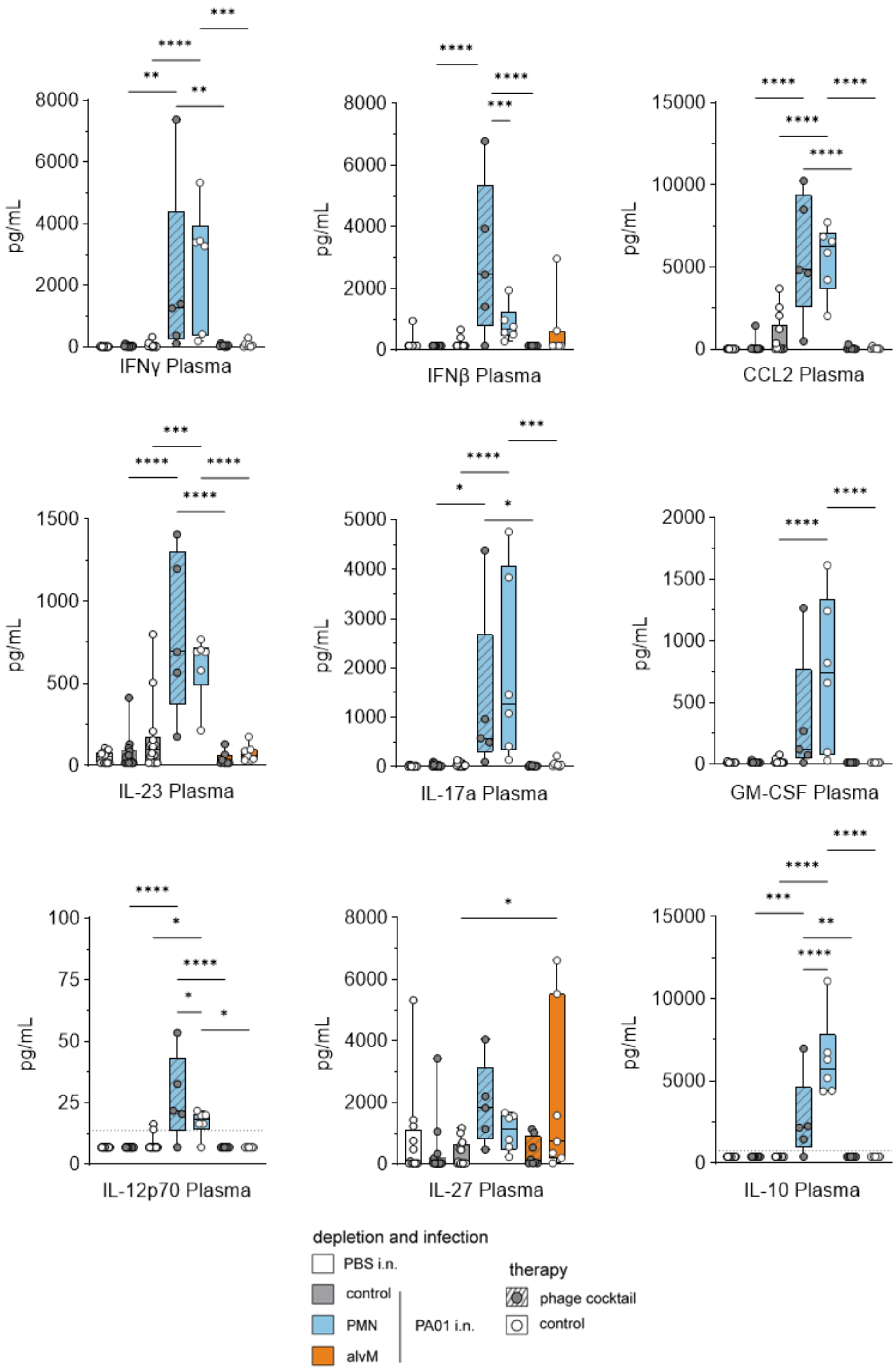

Supplementary Figure S5. Cytokines and Chemokines in plasma of *Pseudomonas*-infected mice. Level of pro-inflammatory cytokines (pg/mL) in plasma of mice analyzed at 24 hpi. Results are shown as box plots depicting median, quartiles, and range, as determined by ordinary one-way ANOVA with Tukey´s multiple comparisons test: *p < 0.05; **p < 0.01; n = 12 control undepleted, sham-infected, control treated mice (white), n = 14 control undepleted, PAO1-infected mice with phage treatment (grey, dashed lines), n = 14 control undepleted, PAO1-infected, control treated mice (grey), n = 5 mice with PMN cell depletion, PAO1-infection and phage treatment (blue, dashed lines), n = 6 mice with

PMN depletion, PAO1-infection and control treatment (blue), n = 8 mice with AlvM depletion, PAO1-infection and phage treatment (orange, dashed lines), n = 7 mice with AlvM cell depletion, PAO1-infection and control treatment (orange). Dotted lines represent the detection limit. BALF, bronchoalveolar lavage fluid; IL, interleukin; CCL2, chemokine (C-C motif) ligand 2; GM-CSF, Granulocyte-macrophage- colony stimulating factor.
